# Therapies in Cervical Cancer—Editorial

**DOI:** 10.3390/cancers15020537

**Published:** 2023-01-16

**Authors:** Raj Naik, Nick Wood, Antonios Anagnostopoulos, Dennis Yiannakis

**Affiliations:** 1 Northern Gynaecological Oncology Centre, Queen Elizabeth Hospital, Gateshead NE9 6SX, UK; 2 Department of Gynaecological Oncology, Lancashire Teaching Hospital, Preston PR2 9HT, UK; 3 Angios Savvas Cancer Hospital, 11522 Athens, Greece; 4 Department of Medical Oncology, Lancashire Teaching Hospital, Preston PR2 9HT, UK

George Papanikolaou is famously quoted as saying “the first observation of cancer cells in the smear of the uterine cervix gave me one of the greatest thrills I ever experienced during my scientific career” [1].

It is with this same “sense of thrill” that we, the guest editors, joyously accepted the invitation to edit this special themed edition of *Cancers* titled “Therapies in Cervical Cancer”.

On contacting the scientific and medical/surgical communities across the globe to ask them to speedily finalise their studies and write up their manuscripts for submission, the same “sense of thrill” became infectious, no less so than as if it was a human papilloma virus!

As expected, the manuscripts and submissions arrived in their multitude.

It is now, with an immense “sense of thrill”, that we present to you 14 publications from a range of investigators from across Europe, USA, and Asia, including research from Spain, Greece, Germany, Czech Republic, Japan, the UK, USA, and France. Of course, we are delighted with such an outstanding international contribution to this Special Edition.

In presenting the publications within this Special Edition, we feel impelled to reference another famous quote, this time from the book *Great Expectations* by Charles Dickens: “Take nothing on its looks; take everything on evidence. There’s no better rule” [2].

With this in mind, we direct the reader to four of the publications in the Special Edition by Wood et al. [3], Leffers et al. [4], Saha et al. [5], and Valasoulis et al. [6]. This collection of scientific studies will provide future investigators with greater insight into novel approaches to treatment, detection, and prevention. Their investigations of Anti-CD40, liquid biopsy methylation, PARP inhibitors, and the HPV vaccine have the potential to become game changers, eradicating the suffering, misery, and pain caused by cervical cancer.

Our sincerest gratitude goes to the team from Prague, led by David Cibula. His pioneering work on sentinel node assessment in cervical cancer has been ground breaking. We are privileged to present two of his studies focusing on: (1) the importance of micro-metastases and ultra-staging in relation to poor survival outcomes [7]; (2) the importance of pathology quality assurance/central pathology reviews in the histological reporting of sentinel nodes [8].

For David and his expert team in the Czech Republic and elsewhere, we are impelled again to reference another famous quote, this time by none other than Aristotle: “intelligence consists not only in the knowledge but also in the skill to apply the knowledge into practice” [9].

It is no surprise that the vociferous debate on the use of minimal-access surgery for performing a radical hysterectomy in early stage cervical cancer is as vibrant now four years on as it was following the publication of the LACC Trial in 2018 [10]. This debate is no less strong with “robotic” surgeons as it is with “straight-stick” surgeons. This Special Edition therefore contains two publications on robotic surgery by Fernandez-Gonzalez et al. [11] and Ponce et al. [12], highlighting quality assurance in robotic surgery and the risk factors associated with poor outcomes. Both publications will lead the international gynaecological oncology surgical community to a more informed, balanced, mature, and consensual position on the surgical management of early stage cervical cancer.

The Charite University Hospital in Berlin is renowned for its incredible contribution to the surgical management of early stage and recurrent cervical cancer, and Mustafa Muallem is to be congratulated for following the strong traditions of his predecessors, Achim Schneider and Christardt Koehler, contributing two publications in this Special Edition that provide an in-depth comprehensive anatomical description of para-metria, para-colpos, and pelvic autonomic nerve systems, as well as providing a detailed description of nerve-sparing radical hysterectomy, including for locally advanced cervical tumours greater than 4 cm in size, as a means to reduce the post-operative neurological damage inflicted to the patient by the procedure’s radicality [13,14].

We also express our greatest respect to our radiation/clinical/medical oncology colleagues. The advances in radiation and medical oncology in the treatment of cervical cancer in recent years have been tremendous. This Special Edition presents two publications by Kokabu et al. [15] and Tomizawa et al. [16], which identify the major advances in three-dimensional image-guided multi-catheter interstitial brachytherapy for bulky IIB-IVB cervical cancer in the pelvic sterilisation of cervical cancer whilst minimising morbidity; additionally, the second publication confirms the utility of the 2018 revised FIGO staging system in making significant advances and contributions to radiation oncology compared with the 2009 version in the era of three-dimensional image-guided brachytherapy.

The final publication in this Special Edition is by Frelaut et al. [17], presenting an international survey involving France, the UK, Netherlands, Israel, and Spain, investigating the current practices of providing gold-standard therapies to older women. Whilst showing that oncologists should treat old fit women as they would young, they recommend modify/downgrading treatment for old unfit women. However, there was a lack of appropriate tools to differentiate the old and fit from the unfit, meaning there is more work needed to improve accurate geriatric evaluations.

It is on this note that we make our final comments: there is “more work to do”.

We hope this Special Edition on Therapies in Cervical Cancer will continue to inspire research communities, whether it be in the laboratory, clinic, or operating theatre, to pursue their aspirations of improving the lives of women affected by cervical cancer boldly and courageously with the same “sense of thrill” as George Papanicolaou.
